# The C-Terminal Tail of Mitochondrial Transcription Factor A Is Dispensable for Mitochondrial DNA Replication and Transcription In Situ

**DOI:** 10.3390/ijms24119430

**Published:** 2023-05-29

**Authors:** Natalya Kozhukhar, Mikhail F. Alexeyev

**Affiliations:** Department of Physiology and Cell Biology, University of South Alabama, Mobile, AL 36688, USA

**Keywords:** GeneSwap approach, mtDNA replication, mtDNA transcription, mtDNA instability, TFAM

## Abstract

Mitochondrial transcription factor A (TFAM) is one of the widely studied but still incompletely understood mitochondrial protein, which plays a crucial role in the maintenance and transcription of mitochondrial DNA (mtDNA). The available experimental evidence is often contradictory in assigning the same function to various TFAM domains, partly owing to the limitations of those experimental systems. Recently, we developed the GeneSwap approach, which enables in situ reverse genetic analysis of mtDNA replication and transcription and is devoid of many of the limitations of the previously used techniques. Here, we utilized this approach to analyze the contributions of the TFAM C-terminal (tail) domain to mtDNA transcription and replication. We determined, at a single amino acid (aa) resolution, the TFAM tail requirements for in situ mtDNA replication in murine cells and established that tail-less TFAM supports both mtDNA replication and transcription. Unexpectedly, in cells expressing either C-terminally truncated murine TFAM or DNA-bending human TFAM mutant L6, HSP1 transcription was impaired to a greater extent than LSP transcription. Our findings are incompatible with the prevailing model of mtDNA transcription and thus suggest the need for further refinement.

## 1. Introduction

Most metazoan cells produce bulk of their ATP through the process of oxidative phosphorylation, which critically depends on 13 polypeptide subunits encoded in mitochondrial DNA (mtDNA), a circular DNA molecule with a typical length in metazoans in the range of 14–20 kbp located in the mitochondrial matrix. The maintenance, expression, and organization of mtDNA depend on mitochondrial transcription factor A (TFAM), a member of the HMGB subfamily of the high mobility group (HMG) proteins.

TFAM affects cellular physiology predominantly through its contributions to mtDNA replication and transcription, which are linked. According to one of the models, a fraction of transcripts from the mitochondrial light strand (L-strand) promoter (LSP, whose transcripts are identical in sequence to mtDNA heavy strand [H-strand]) are prematurely terminated at the guanine-rich conserved sequence block II (CSBII) to generate primers for mitochondrial H-strand replication [1,2,3,4,5]. Indeed, knockouts (KO) for either POLRMT or TFB2M, two other key players in mitochondrial transcription, resulted in the loss of mtDNA, underscoring the critical link between mtDNA transcription and replication [6].

In vivo, the whole-body TFAM knockout (KO) is embryonically lethal and is accompanied by severe mtDNA depletion [7]. In contrast, tissue-specific TFAM KOs have variable phenotypes, some of which are relatively mild [8,9,10]. These mild phenotypes are particularly perplexing, considering that TFAM KO in situ results in the loss of mtDNA, which then results in the inability to produce ATP through oxidative phosphorylation, the main pathway for ATP production in most tissues [6].

Mammalian TFAMs conform to the overall domain architecture of HMGB proteins. As mitochondrial proteins, in addition to the “classical” two HMG-B boxes, linker and tail domains, they also contain an N-terminal cleavable matrix targeting sequence (MTS). This MTS is characterized by variable length and no conservation at the primary amino acid (aa) sequence level between orthologs. Remarkably, putative MTSs of most tested TFAM orthologs are at least partially functional in human cells, including the very short 11 aa MTS from *Callorhinchus milii* TFAM [6].

**Figure 1:**
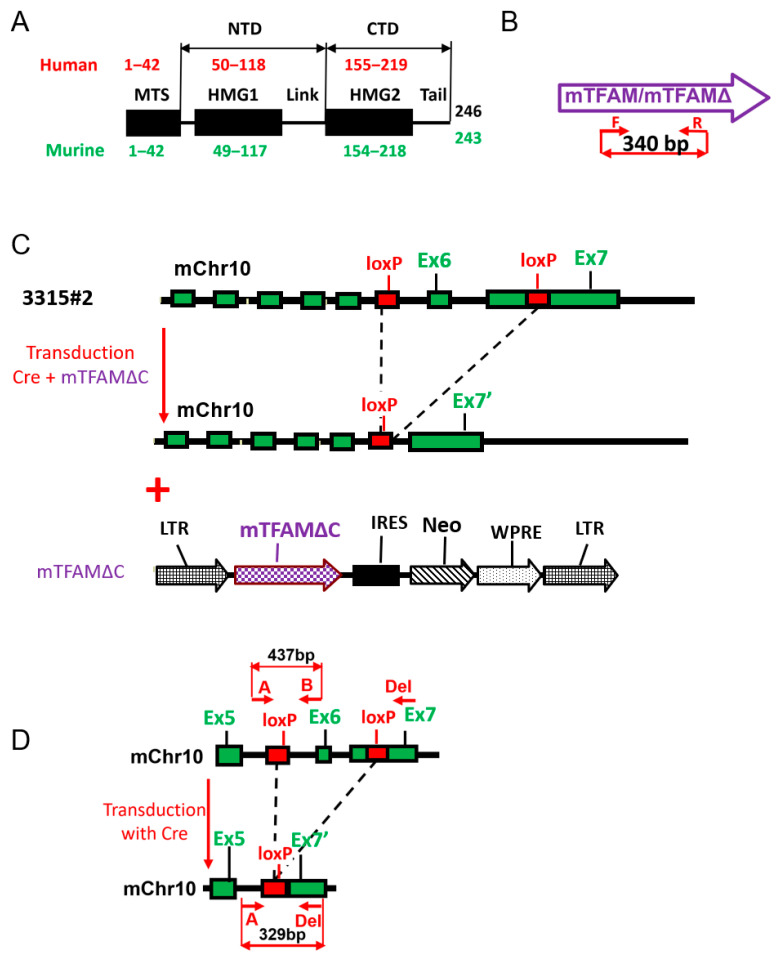
GeneSwap in murine cells. (**A**) The domain structure of the murine TFAM in relationship to hTFAM. (**B**) A diagram of PCR genotyping strategy of the introduced mTFAMwt or mTFAMΔ cDNAs. (**C**) A schematic diagram of the TFAM GeneSwap in mouse cells. Ex, exon. Ex7′ = truncated exon 7. (**D**) A diagram of PCR genotyping strategy of the Cre-mediated deletion of Exons 6 and 7 in mouse chromosome 10. Primers are as per Table S1.

TFAMs may also possess a leader sequence of variable length located between the MTS and the first HMGB box (HMG1, also known as HMG Box 1, also known as HMG Box A, Figure 1A).

TFAMs bind mitochondrial DNA (mtDNA) both sequence-specifically and nonspecifically. In mammalian cells, sequence-specific binding occurs upstream of LSP and mitochondrial H-strand promoter 1 (HSP1) and produces specific DNase I footprints [11,12]. Importantly, at HSP1, TFAM binds in reverse orientation compared to LSP [11,12,13]. Crystal structures show that TFAM binding at LSP, HSP1 or nonspecific DNA (NSP) induces sharp (~180°) bends, thus facilitating mtDNA compaction and assembly into nucleoids [13,14,15,16], structures where mtDNA replication and transcription are thought to occur (however, in a number of studies nucleoids with little or no TFAM were observed [17,18,19,20,21]). It has been suggested that TFAM bending at LSP positions the TFAM tail near the transcriptional machinery and therefore is necessary for the full activation of transcription. This notion is consistent with the observation that TFAM mutants in DNA bending are defective in vitro in initiating transcription at LSP but fully active at HSP1 transcription [13,14]. Conversely, it has been proposed that reverse binding at HSP1 places TFAM’s tail in the proximity of the transcriptional machinery without the need for DNA bending for transcription activation [14].

Recently, through a multitude of in vivo, in vitro, and in situ studies, a holistic picture of the functional significance of TFAM domains have begun to emerge. Thus, isolated HMG1 has DNA-binding properties, whereas the second HMG box (HMG2, also known as HMG Box 2 and HMG Box B) is unable to bind DNA on its own [22]. However, HMG2 plays the leading role in determining TFAM species specificity [6]. TFAM exists as a monomer in solution but dimerizes upon DNA binding [22]. It has been suggested that either HMG2 [23] or the C-terminal tail [24] is responsible for TFAM dimerization. However, the structure of the hTFAM–DNA complex indicates that the dimer interface may lie within HMG1 [13].

The function of the TFAM C-terminal tail is of particular interest to us, primarily because of the considerable debates surrounding it. Unlike other HMGB proteins’ C-terminal tails that are negatively charged [25], the tails of most TFAMs have a net positive charge. Curiously, the tails of some TFAM orthologs that are functional in human mtDNA replication (e.g., TFAMs from the cat, mouse, pig, and pika [6]) are charged negatively. Moreover, hTFAM can substitute for murine TFAM (mTFAM) in transgenic animals [26]. Collectively, these observations suggest that TFAM’s function may not critically depend on the charge of its C-terminus. However, an earlier study suggested the importance of the negative charge of the TFAM tail for transcription from LSP [11].

It has been shown that the last 10 aa of the TFAM tail are dispensable for transcription from the LSP promoter in vitro. However, the deletion of 15 or more residues severely impaired transcription [11,27,28]. TFAM tail has been suggested to interact with and recruit another mitochondrial transcription factor, TFB2M [29], and mitochondrial RNA polymerase (POLRMT) [30]. These observations led to the notion of the critical role played by the TFAM tail in mitochondrial transcription.

Removal of the tail reduced the affinity of TFAM for LSP, mitochondrial termination-associated sequence, and NSP by 1034-, 825-, and 653-fold, respectively [31]. Since the loss of the tail affected TFAM’s affinity for LSP the most, these observations suggested that the tail may be involved in promoter selection (sequence-specific DNA binding [22]) and nucleoid organization [31]. However, another study established that the deletion of the tail reduced TFAM affinity to LSP by only approximately two-fold and that tail-less TFAM binds NSP DNA with near wild-type affinity [22].

The role of the TFAM tail in mtDNA replication remains incompletely defined. A seminal study by Matsushima et al. established that chicken TFAM retaining only the three tail aa most proximal to HMG2 retains full activity in mtDNA replication in TFAM-haploinsufficient DT40 cells. As a caveat, that study reported that the TFAM tail contains both stimulatory and inhibitory sequences for mtDNA replication [32]. Similarly, tail-less TFAM rescued mtCN in TFAM-depleted HeLa cells, indicating that the TFAM tail may be dispensable for mtDNA replication [28,33]. However, conclusions in these studies were drawn from examining cells in which both wild-type and tail-less TFAMs were coexpressed, and therefore the contribution of complex interactions between these two forms could not be excluded.

Here, we report the reverse genetic analysis of the TFAM tail using the recently developed GeneSwap approach [6]. The GeneSwap approach allows replacing, in cultured cells, the wild-type cDNA with a cDNA encoding mutant protein. Therefore, the interpretation of the outcomes of GeneSwap experiments is not confounded by the coexpression of the wild-type polypeptides. Additionally, being an in situ technique, the GeneSwap approach differs from in vitro methods in that it examines the transcription and replication of a native mix of topological isoforms of full-length mtDNA molecules using a native complement of mitochondrial proteins.

## 2. Results

Recently, we demonstrated that the cultured human cells knocked out (KO) for the genes encoding the key proteins involved in mtDNA replication are viable and can be cultivated in high glucose media supplemented with uridine and pyruvate. This observation laid the groundwork for developing the GeneSwap approach, a method for reverse genetic analysis of mtDNA replication proteins [6]. This method allows for more rigorous testing of C-terminal tail requirements, as the testing is conducted in the absence of input from wild-type (wt) protein.

### 2.1. The Tail Domain of mTFAM Is Dispensable for mtDNA Replication

Previous studies indicated that 25 C-terminal aa of hTFAM are required for in vitro transcription from the LSP promoter. Considering that transcription from this promoter is believed to contribute a primer for mitochondrial H-strand replication [1,2,3,4,5], the tail-less hTFAM should be compromised in mtDNA replication due to the inability to initiate DNA synthesis at the origin of the H-strand replication (Ori_H_). However, the available experimental evidence suggests that the TFAM tail may be dispensable for mtDNA replication [28,33]. The experimental limitations can potentially explain this apparent contradiction (either the reductive nature of in vitro transcription systems or the coexpression of the wild-type and mutant TFAMs in previous experiments in situ). Since the GeneSwap approach is free of either limitation, we applied it here.

To our knowledge, TFAM tail requirements for mtDNA replication have not been assessed for mTFAM. Therefore, we set up a TFAM GeneSwap system in murine cells. Mouse embryonic fibroblasts (MEFs) from TFAM^loxP/loxP^, Gt(ROSA)26Sor^+/lox-Stop-lox-mito-YFP^ animals were kindly provided by Nils-Goran Larsson [34]. In these cells, exons 6 and 7 and the polyA site of mTFAM were flanked by loxP sites (Figure 1C,D) [7]. In addition, a mitochondrially targeted EYFP gene preceded by a terminator cassette flanked by loxP sites was inserted into the ROSA26 locus [34]. In the presence of the Cre recombinase, TFAM exons 6 and 7 were lost, and expression of the mitochondrially targeted EYFP was induced. We immortalized these MEFs with a retrovirus encoding SV40 large T-antigen [35] to generate the 3315#2 cell line, which was suitable for GeneSwap.

**Figure 2:**
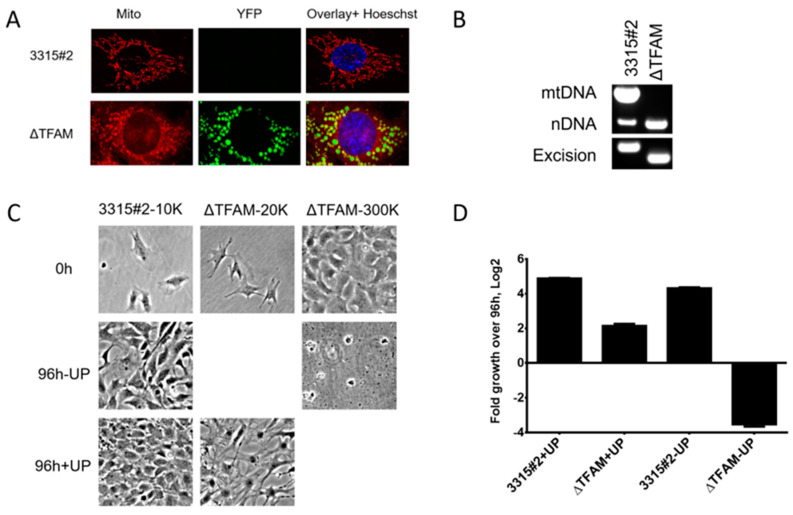
TFAM inactivation in 3315#2 murine fibroblasts. (**A**) mTFAM inactivation is accompanied by mitochondrial expression of EYFP because of the simultaneous excision of the terminator cassette preceding the mitochondrially targeted EYFP gene in the ROSA26 locus. (**B**) TFAM inactivation is accompanied by the loss of mtDNA. The 3315#2 cells were transduced with adenovirus encoding Cre recombinase (Ad5CMVCre), and EYFP-positive clones were collected via FACS and plated in +UP medium. The resulting colonies were genotyped via PCR for the presence of mtDNA and deletion of TFAM exons 6 + 7 using the primers listed in Table S1. (**C**,**D**) TFAM deletion is accompanied by the loss of viability in -UP medium. Cells were seeded at 1 × 10^4^ (10K), 2 × 10^4^ (20K), or 3 × 10^5^ (300K) per well of the 6-well plate, allowed to attach overnight, photographed, and counted (3 wells per cell density). The remaining wells received either +UP or -UP media and were incubated for an additional 96 h, after which photographing and counting were repeated. Representative results of two independent experiments, with triplicate measurements per experimental condition. Means ± SDs.

The 3315#2 cell line was validated by inducing TFAM deletion with adenovirus-encoded Cre recombinase and examining the phenotype of the resulting cells. Introduction of the Cre recombinase into 3315#2 cells activated mitochondrial expression of EYFP (Figure 2A) and induced the deletion of mTFAM exons 6 + 7 and the loss of mtDNA (Figure 2B). Of note, mitochondrial enlargement and rounding in TFAM^−/−^ 3315#2 cells were much more pronounced than previously reported for neurons [34]. The resulting ρ^0^ cells grew well in the +UP medium but did not survive in the -UP medium (Figure 2C,D).

**Figure 3:**
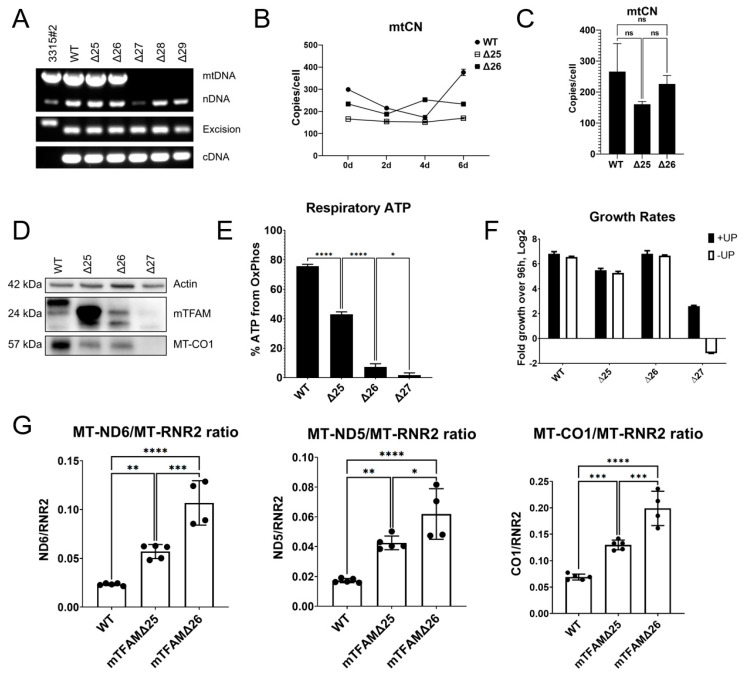
Effects of the C-terminal mTFAM truncations. (**A**) 26 C-terminal aa residues of mTFAM are dispensable for mtDNA replication. Genotyping as per diagrams in Figure 1C,D using primers listed in Table S1. B, C, C-terminal truncations did not result in a significant reduction in mtCN. A time course of mtCN over 6 days. (**B**) Most error bars are smaller than the symbols. Representative results of two independent experiments. (**C**) Cumulative mtCN data over the four time points in B. One-way ANOVA with post hoc Tukey’s test. ns, not significant. (**D**) Expression of truncated mTFAM variants and MT-CO1 in GeneSwapped cells. A representative of two independent experiments. Calculated molecular weights of polypeptides are presented in kilodaltons. (**E**) % ATP derived from OXPHOS in GeneSwapped cells with various mTFAM variants. Representative results from two independent experiments with at least triplicate measurements per experimental condition. One-way ANOVA with post hoc Tukey’s test. *, *p* < 0.05; ****, *p* < 0.0001. (**F**) Growth of GeneSwapped cells expressing various mTFAM variants in either +UP or -UP media. A representative of two biological experiments each with three technical replicas. Note that cells expressing the mTFAMΔ27 variant fail to grow in -UP medium, which is consistent with their ρ^0^ status. (**G**) Steady-state levels of the MT-RNR2 transcript (HSP1 promoter) are affected the most by TFAM truncations. Cumulative results of at least four biological experiments, each with at least two technical replicates. Mean ± SD. One-way ANOVA with Post hoc Tukey’s test. *, *p* < 0.05; **, *p* < 0.01; ***, *p* < 0.001; ****, *p* < 0.0001.

Analysis of the C-terminal mTFAM truncations revealed that the loss of as many as 26 C-terminal amino acids did not impair mtDNA replication by mTFAM (Figure 3A).

mtCN in cells expressing wt mTFAM was unstable over the examined 6-day cultivation period, which contributed to the lack of statistical significance between mtCNs in all analyzed cell lines (Figure 3B,C). This was despite the fact that the mTFAMΔ26 variant was expressed at a lower level than either wt mTFAM or mTFAMΔ25 (Figure 3D). Unlike mtCN, there was a strict correlation between the percentage of ATP produced through respiration and the extent of the C-terminal deletion (Figure 3E). In accordance with mtDNA maintenance, mTFAMΔ25 and mTFAMΔ26, but not mTFAMΔ27, grew in -UP media (Figure 3F). Analysis of HSP1, HSP2, and LSP transcription in cells expressing truncated mTFAMs suggested that, as with hTFAM, C-terminal deletions affected HSP1 transcription the most. This was evidenced by the increased steady-state abundance of MT-ND6, MT-ND5, and MT-CO1 transcripts relative to MT-RNR2 (Figure 3G).

Similarly, deletion of up to 23 C-terminal aa in hTFAM did not ablate mtDNA replication or transcription in human cells, and HSP1 transcription was affected to a greater extent than LSP transcription (Figure S1).

### 2.2. TFAM DNA-Bending Deficiency Predominantly Impairs HSP1 Transcription

The finding that HSP1 transcription is impaired to a greater extent than LSP transcription in cells expressing tail-less TFAM was unexpected, considering that in the prevailing model, the C-terminal tail plays the same role at both promoters: recruitment of the transcription machinery. Furthermore, evidence suggests that the TFAM tail may contribute to DNA bending [36], and therefore, tail-less TFAM would be expected to be more impaired in LSP transcription, which requires DNA bending for the proper assembly of the transcription apparatus in this model. Therefore, we were interested in the contribution of DNA bending to LSP and HSP1 transcription in situ.

**Figure 4:**
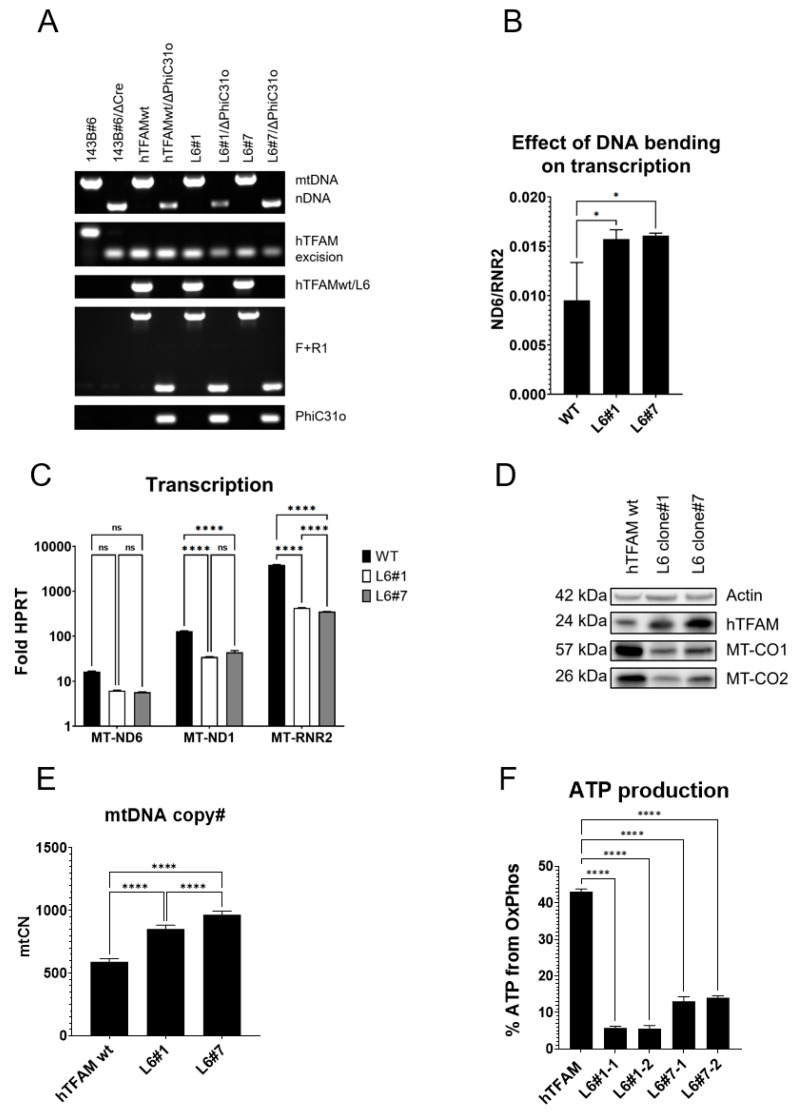
Effects of the hTFAM DNA bending defect. Diagrams for PCR-genotyping strategies for validating of substitution of hTFAMwt and hTFAM L6 for hTFAMwt in 143B#6 cells by PhiC31o-mediated excision is presented in Figure S2. Primers as per Table S1. (**A**) The hTFAM L6 bending mutant supports hmtDNA replication. Primers as per Table S1. hTFAM excision as per Figure S2A. hTFAMwt/L6 as per Figure S2B. F + R1 as per Figure S2C. PhiC31o as per Figure S2D. (**B**) Increased ratio of steady-state levels MT-ND6/MT-RNR2 indicates predominant suppression of the HSP1 promoter in cells expressing hTFAM L6 bending mutant. A composite of three independent experiments with at least three technical replicates per experimental condition. *, *p* < 0.05. One-way ANOVA with post hoc Tukey’s test. (**C**) Effect of DNA bending defect on transcript levels from individual promoters. Representative results of two independent experiments. Ordinary two-way ANOVA with Tukey’s multiple comparisons test, with a single pooled variable. ****, *p* < 0.0001; ns, not significant. (**D**) Effect of hTFAM bending defect on the expression of mtDNA-encoded polypeptides. Calculated molecular weights of polypeptides are presented in kilodaltons. (**E**) mtCN is slightly elevated in clones expressing the hTFAM L6 bending mutant. Representative results of two independent experiments with four measurements per experimental condition. One-way ANOVA with post hoc Tukey’s test. (**F**) ATP production through respiration is suppressed in cells expressing the hTFAM L6 bending mutant. Representative results of two independent experiments with at least three replicate measurements per experimental condition. One-way ANOVA with post hoc Tukey’s test.

Ngo et al. extensively characterized the TFAM bending mutant, L6 [14]. In this mutant, six basic aa in the linker, which are critical for DNA charge neutralization during DNA bending, were substituted with alanines. We substituted wt hTFAM cDNA in 143B#6 cells [6] with cDNA encoding the L6 mutant of hTFAM, and validated two independent clones (#1 and #7) by PhiC31o-mediated excision of the L6 mutant cDNA as per diagrams presented in Figure S2. In both cases, excision of the L6 mutant with PhiC31o recombinase resulted in the loss of mtDNA (Figure 4A). Therefore, neither clone expresses any other functional TFAM isoform apart from the L6 mutant. We further examined mitochondrial transcription, mtCN, expression of TFAM and mtDNA-encoded respiratory subunits, and respiration in both clones.

Unexpectedly, in both clones, expression of the DNA-bending TFAM mutant resulted in a greater depression of HSP1 transcription than LSP transcription (Figure 4B). This finding is not in agreement with the prevailing model of mitochondrial transcription, which posits that DNA bending is required for LSP but not HSP1 transcription [13,14,16].

In both clones, hTFAM L6 was expressed at a greater level than hTFAM wt in a control clone. Nevertheless, mtDNA-encoded transcripts (Figure 4C) and their translation products (Figure 4D) were less abundant in cells expressing the bending mutant, supporting the notion of a generalized mitochondrial transcription defect mediated by hTFAM L6 (note that the reduction in ND6 transcript level was not statistically significant).

This notion is not in agreement with in vitro studies, which established that the L6 mutant retains 100% transcriptional activity at HSP1 [13,14].

In contrast, mtCNs were moderately elevated in cells expressing hTFAM L6 (Figure 4E), roughly reflecting the expression of hTFAM variants (Figure 4D). Finally, cells expressing hTFAM L6 were more glycolytic than cells expressing hTFAM wt (Figure 4F) despite elevated mtCNs, which is yet another example of the genetic separation of TFAM contributions to mtDNA replication and mitochondrial respiratory chain biogenesis [37].

## 3. Discussion

The issue of specific mode(s) of mtDNA replication remains unresolved. Historically, the best understood and most widely accepted strand-asynchronous model of mtDNA replication posits that a fraction of LSP transcripts are terminated prematurely and prime H-strand replication [1,2,3,4,5]. This model predicts that the ablation of LSP transcription would ablate mtDNA replication. In vitro, tail-less TFAM is unable to initiate transcription at the LSP promoter [11,27,28]. Additionally, mitochondrial import of the tail-less TFAM fails to stimulate transcription [38]. Therefore, the strand-asynchronous model of mtDNA replication would predict that tail-less TFAM would be impaired in mtDNA replication. In contrast, several in situ studies have suggested that the TFAM tail may be dispensable for mtDNA replication [28,32,33]. This apparent contradiction can be resolved by either invoking alternative models of mtDNA replication or by pointing out the experimental limitations of the two conflicting groups of experiments. Indeed, in situ experiments were performed in the presence of residual full-length TFAM, whereas in vitro experiments are confounded by the fact that they were performed using nonphysiological templates (short linear DNA fragments, whereas mtDNA is a long linear supercoiled molecule). To illustrate the latter point, more recent in vitro experiments that utilized a larger template indicated that tail-less TFAM might be only slightly impaired in LSP transcription [39]. Moreover, recent structural studies suggest that the assembly of the mitochondrial transcriptional apparatus might be mediated by TFAM recruiting POLRMT through HMG2 interaction with the N-terminal ‘tether’ helix of POLRMT [40]. Therefore, in this study we aimed to resolve the existing controversy by implementing the recently developed GeneSwap approach, which is devoid of the limitations intrinsic to previously used experimental techniques.

The question of whether TFAM’s tail is dispensable for mtDNA replication is inseparable from the question of how to define this tail. Conceptually, TFAM’s tail is a stretch of amino acids downstream of HMG2. Unfortunately, the existing body of literature lacks consensus on the expanse of HMG2. Indeed, in two side-by-side papers on the crystal structure of hTFAM–DNA complexes, TFAM’s tail was assigned either aa 223–246 (24 aa, [14]) or aa 226–246 (21 aa, [16]) in contrast to the “classical” 25 aa tail [11]. To further complicate the issue, the Conserved Domain Database [41,42] assigns aa 135–234 to HMG2 (and therefore the tail is only 12 aa long, aa 235–246), and UniProt [43] assigns the C-terminal tail aa 220–246. In this study, we used human hTFAM (hTFAM) domain boundaries as annotated in UniProtKB Q00059 and corresponding annotation in murine TFAM (mTFAM) UniProtKB P40630 (Figure 1A). In these annotations, TFAM tails are the longest, and therefore conclusions about TFAM tail requirements for mtDNA transcription and replication are the most conservative. Moreover, since HMG domains are homology domains, their boundaries should be defined by protein alignments rather than any alternative means.

Here, we show that C-terminal 26 aa in mTFAM are dispensable for mtDNA transcription and replication. Since the longest annotated mTFAM C-terminus is 25 aa long, our observations indicate that it is dispensable for mtDNA replication. Importantly, C-terminal truncations in mTFAM affected HSP1 transcription to a greater extent than transcription from LSP or HSP2. This observation independently confirms a greater reliance of HSP1 transcription on TFAM’s tail compared to LSP and HSP2. The exact mechanism of this heightened reliance remains to be determined.

The prevailing model for mitochondrial transcription posits that TFAM binds at LSP with its tail protruding in the direction away from the transcription initiation site. Since the tail is thought to bind and recruit TFB2M, this orientation is not conducive to transcription initiation unless TFAM bends mtDNA at the promoter 180°. In contrast, at HSP1, TFAM binds in the opposite orientation with its tail toward the transcription initiation site, which makes HSP1 transcription independent of DNA bending [13,14]. It has been reported that the TFAM tail contributes to DNA bending [36], and therefore this model predicts that tail-less TFAM will be severely impaired in supporting LSP transcription, more so than HSP1 transcription. Furthermore, if proper positioning of the TFAM tail is critically required for transcription initiation, as suggested by this model, then one would expect a severe impairment or even ablation of mitochondrial transcription in cells expressing tail-less TFAM. Here, we established that the tail domain of mTFAM is dispensable for transcription. Furthermore, as reported by steady-state levels of MT-RNR2 and MT-ND6 transcripts, C-terminal truncations suppressed HSP1 transcription to a greater extent than LSP transcription. The prevailing model does not easily explain this finding. Finally, in cells expressing the L6 mutant of hTFAM defective in DNA bending, HSP1 transcription was also impaired to a greater extent than LSP transcription. This finding directly contradicts the notion that DNA bending makes a critical contribution to LSP transcription while being dispensable for HSP1 transcription. Therefore, our findings challenge the existing model of mitochondrial transcription.

Finally, in this study, elevated mtCNs in cells expressing the hTFAM-bending mutant contrasted with impaired expression of mtDNA-encoded polypeptides and a reduced fraction of ATP generated through respiration. This observation demonstrates that TFAM’s contributions to mtDNA replication and respiratory chain biogenesis are genetically separable [37].

How can our results be reconciled with the existing body of experimental evidence, which suggests that LSP transcription depends on TFAM’s tail to a greater extent than HSP1 transcription? We believe that diametrically opposed observations made in some previous studies could be due to the limitations of in vitro systems compared to the GeneSwap approach implemented in this study. Indeed, most in vitro transcription systems are reductionist, lack many components available in situ, and their ionic composition differs from that in situ. Additionally, they utilize short linear DNA fragments encompassing either the LSP or HSP1 promoter, whereas in situ transcription occurs off the ~16,000 base-pair-long, circular mtDNA molecules, many of which are supercoiled. In contrast, the GeneSwap approach studies mtDNA transcription directly in situ and therefore is devoid of these limitations. In support of this line of reasoning, an in vitro system that used a DNA template encompassing both LSP and HSP1 promoters (and therefore more closely reflecting an actual transcription template) reported that tail-less hTFAM-stimulated transcription occurs more efficiently at LSP than at HSP1, which is consistent with our findings [39]. Moreover, Morozov et al. were unable to detect TFAM–TFB2M interactions in vitro by crosslinking assays. Instead, TFAM crosslinked with the N-terminal portion of POLRMT, predominantly through its C-terminus [30]. Similarly, Posse et al. suggested that TFAM C-terminus may recruit POLRMT rather than TFB2M [44]. Our data appear to support this latter model in which TFAM recruits the transcription machinery through its interactions with POLRMT rather than with TFB2M, except for the critical role of the TFAM tail in the recruitment. Finally, crystal structures of open transcription complexes at LSP and HSP suggest that TFAM binds at these promoters in the same orientation [40], which is a dramatic reversal of the previously reported crystallographic and in vitro data [12,13,14,16,45]. Our data do not agree with this model, since this model predicts similar sensitivity of promoters to TFAM bending defects and higher sensitivity of LSP to TFAM C-terminal truncations.

## 4. Materials and Methods

### 4.1. Common Procedures

The GeneSwap approach [6] as well as methods for cell cultivation [6,46], retrovirus generation [6,47], quantitation of mitochondrial transcripts, mtCN determination by dddPCR [47,48], and analysis of cellular respiration [6,37] were described in our previous manuscripts and are incorporated here by reference.

### 4.2. Cells

Mouse embryonic fibroblasts TFAM^loxP/loxP^, Gt(ROSA)26Sor+/lox-Stop-lox-mito-YFP were kindly provided by Nils-Goran Larsson [34]. They were immortalized with SV40 large T-antigen using retrovirus rv.3315 [35], resulting in the 3315#2 cell line. The PCR profile of this clone with primers specific to mouse amelogenin is consistent with the female sex. Primers for PCR genotyping of the TFAM^loxP/loxP^ and TFAM^−/−^ alleles are listed in Table S1. Genotyping was performed according to the schemes presented in Figure 1D using the protocol described in [49].

The 143B#6 cell line was described previously [6].

Phoenix Ampho cells were obtained from the National Gene Vector Biorepository.

### 4.3. DNA Constructs

Plasmids were constructed according to standard recombinant DNA protocols [50]. C-terminal truncations were introduced via PCR. Point mutations were generated using overlap extension PCR [51]. Nucleotide sequences of TFAM variants used in this study are listed in Table S2. Mutant mTFAMs were cloned into pMA3883 (Figure S3), whereas mutant hTFAMs were cloned into pMA3448rc or pMA4659 (Figure S3).

### 4.4. Growth Rates

Growth rates were established over 96 h period in either permissive (+UP) or nonpermissive (-UP) media. Cell counts were determined in triplicate wells immediately after attachment (0 h time point) and after 96 h growth in the indicated media (96 h time point) using a Beckman–Coulter Z1 particle counter. The results are presented as log2 (cell count at 96 h/cell count at 0 h).

### 4.5. Adenovirus Encoding Cre Recombinase

(Ad5CMVCre) was supplied by the University of Iowa Viral Vector Core Facility.

### 4.6. GeneSwap

GeneSwap in 143B#6 cells was executed as described earlier [6]. GeneSwap in murine cells was executed as in Figure 1C. To implement GeneSwap, 143B#6 or 3315#2 cells were cotransduced with rv.3442 and a retrovirus carrying an hTFAM or mTFAM variant. This resulted in the simultaneous excision of the endogenous hTFAMwt (or deletion of exon 6 and partial deletion of exon 7 of mTFAM) and the introduction of either wt or mutant cDNA.

### 4.7. Quantification of Mitochondrial Transcripts

Quantitation of mitochondrial transcripts was performed via RT–qPCR using the primers listed in Table S1. RNA was isolated using an EZ-10 DNAaway RNA Miniprep Kit (Bio Basic, Amherst, NY, USA, Cat# BS88136) and treated with a gDNA removal kit (Enzo Life Sciences, Farmingdale, NY, USA, Cat# ENZ-KIT136-0050) to reduce mtDNA contamination prior to reverse transcription with a SensiFast cDNA synthesis kit (Bioline USA, Taunton, MA, USA, Cat# BIO-65053), which was supplemented with primers for MT-ND6 RT–qPCR. In most experiments, three transcripts representative of three mitochondrial promoters were quantitated using a SYBR Fast kit (Roche Holdings AG, Basel, Switzerland, Cat# KK4601): MT-ND6 (for the light strand promoter, LSP), MT-RNR2 (for the heavy strand [H-strand] promoter 1, HSP1), and MT-ND1 (for the H-strand promoter 2, HSP2). Some experiments also included the quantification of MT-CO1 transcripts. Transcript abundancies were normalized to those of hypoxanthine phosphoribosyltransferase 1 (HPRT1) for human cells and to glyceraldehyde-3-phosphate dehydrogenase (Gapdh) for murine cells. To quantify retrovirally expressed C-terminally truncated hTFAM transcripts, primers were designed across the vector-cDNA junction.

### 4.8. Western Blotting

Western blotting was performed as described previously [52]. The antibodies used were anti-MT-CO1 (Abcam, Cambridge, MA, USA, Cat#ab14705), anti-MT-CO2 (Abcam, Cambridge, MA, USA, Cat#ab91317), anti-m + hTFAM (PhosphoSolutions, Aurora, CO. Cat#2001-TFAM), anti-hTFAM (Abclonal, Woburn, MA, USA, Cat#A3173), and anti-β-actin (Sigma, St. Louis, MO, USA, A5441).

### 4.9. Amino Acid Alignments

Amino acid alignments were derived using the MUSCLE algorithm of the MegAlign Pro program Lasergene 17 package (DNASTAR Inc., Madison, WI, USA).

### 4.10. Statistical Analyses

Statistical analyses were performed using the GraphPad Prism v.9.1.0 software package.

## 5. Conclusions

Here, we used the recently developed GeneSwap technique to establish, at a single aa resolution, the TFAM tail requirements for in situ mtDNA replication in murine and human cells. Our data conclusively demonstrate that the mTFAM tail is dispensable for mtDNA replication and transcription. They are also consistent with the dispensability of the hTFAM tail. We show that TFAM C-terminal truncations have a greater effect on HSP1 transcription than on LSP transcription. Similarly, TFAM bending defect had a greater effect on HSP1 transcription. These three observations are not easily explainable by the current models of mitochondrial transcription that postulate the critical role of the TFAM C-terminus in the recruitment and assembly of the transcription machinery, recognition of LSP but not HSP1, or predict a greater effect of TFAM truncations and bending defects on LSP transcription than on HSP transcription. Therefore, we suggest that the current model of mitochondrial transcription needs to be further refined to accommodate these new findings as well as the findings of Uchida et al. [39], who used an improved in vitro assay to independently find a similar discrepancy in the relative sensitivity of LSP and HSP1 transcription to TFAM truncations.

## Data Availability

The data presented in this study are contained in the article and supplementary material.

## Supplementary Materials

The supporting information can be downloaded at: https://www.mdpi.com/article/10.3390/ijms24119430/s1.
