# Supplementary material for: The C-Terminal Tail of Mitochondrial Transcription Factor A Is Dispensable for Mitochondrial DNA Replication and Transcription In Situ
Source: Int J Mol Sci. 2023 May 29;24(11):9430. doi: 10.3390/ijms24119430 (PMC10253692; doi:10.3390/ijms24119430)
Supplement: Supplementary file 1 [file ijms-24-09430-s001.zip › IJMS Supplementary figures.pdf]

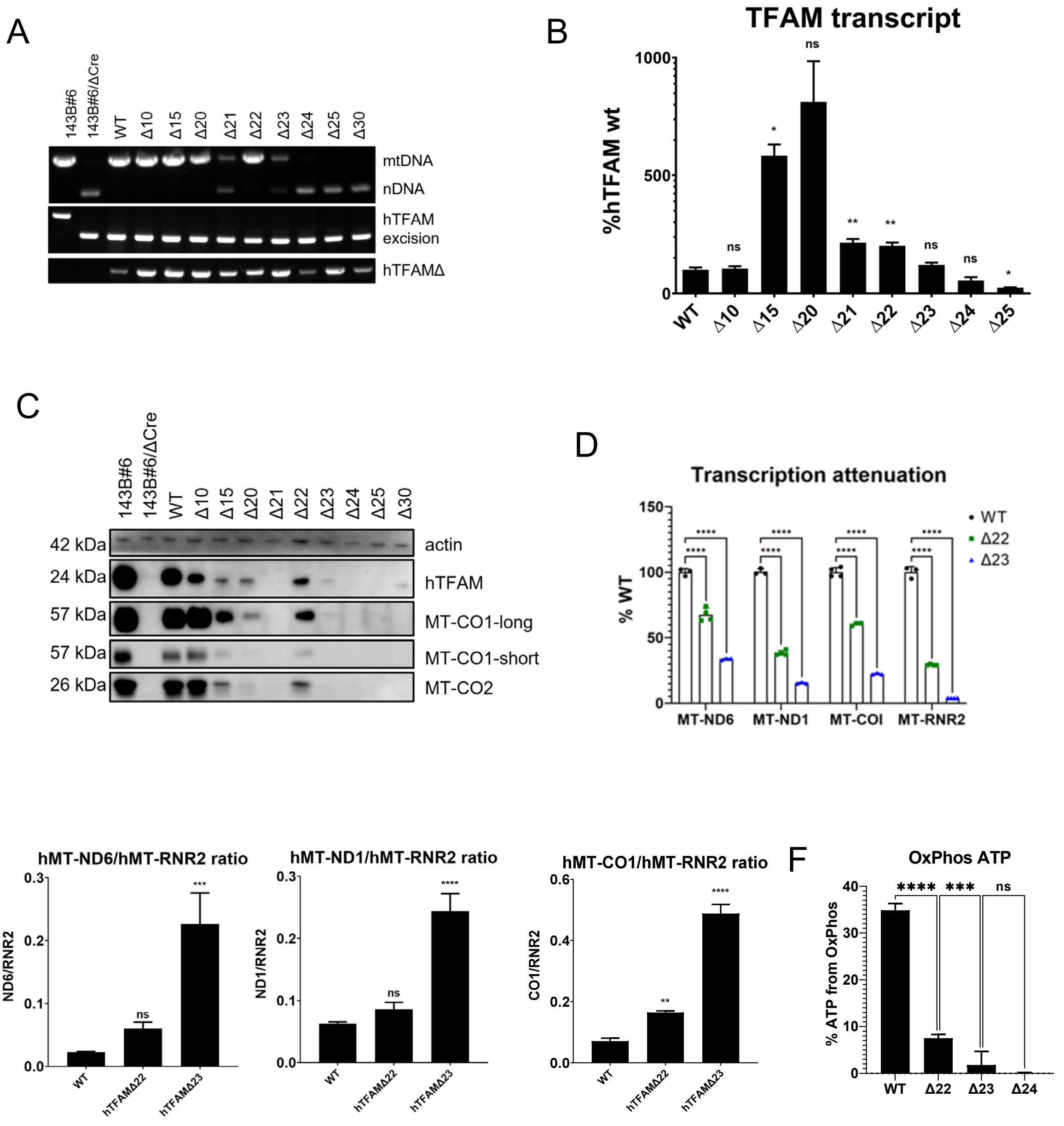

Figure S1. 23 C-terminal aa of the hTFAM are dispensable for mtDNA replication. A, mtDNA retention in cells expressing C-terminally truncated hTFAM variants. Please, note that due to the differences in vector backbone sequences, selected primers do not amplify wt hTFAM in 143B#6 cells. B, hTFAM transcript expression in cells expressing wt and truncated hTFAM variants. One-way ANOVA. .*, P<0.05; **, P<0.01, ns, not significant. C, expression of the mtDNA-encoded polypeptides is in agreement with the expression of truncated TFAMs but does not correlate with levels of TFAMΔ transcripts. MT-COI short and MT-COI long, short and long exposures. D, Transcription attenuation in cells expressing hTFAMΔ22 and hTFAMΔ23 variants. A representative of three independent experiments. Two-way ANOVA. ****, P<0.0001. E, hTFAM C-terminal truncations have the greatest effect on HSP1 transcription. Transcript ratios were determined by $2^{-\Delta\Delta Ct}$ method. Means ± SD. One-way ANOVA with post-hoc Tukey test. **, P<0.01; ***, P<0.001; ****, P<0.0001; ns, not statistically significant. Significance levels compared to wt. F, The fraction of ATP generated through OXPHOS is reduced in cells expressing hTFAM Δ22 and Δ23 variants. Means ± SD. One-way ANOVA with post-hoc Tukey test. ***, P<0.001; ****, P<0.0001; ns, not statistically significant.

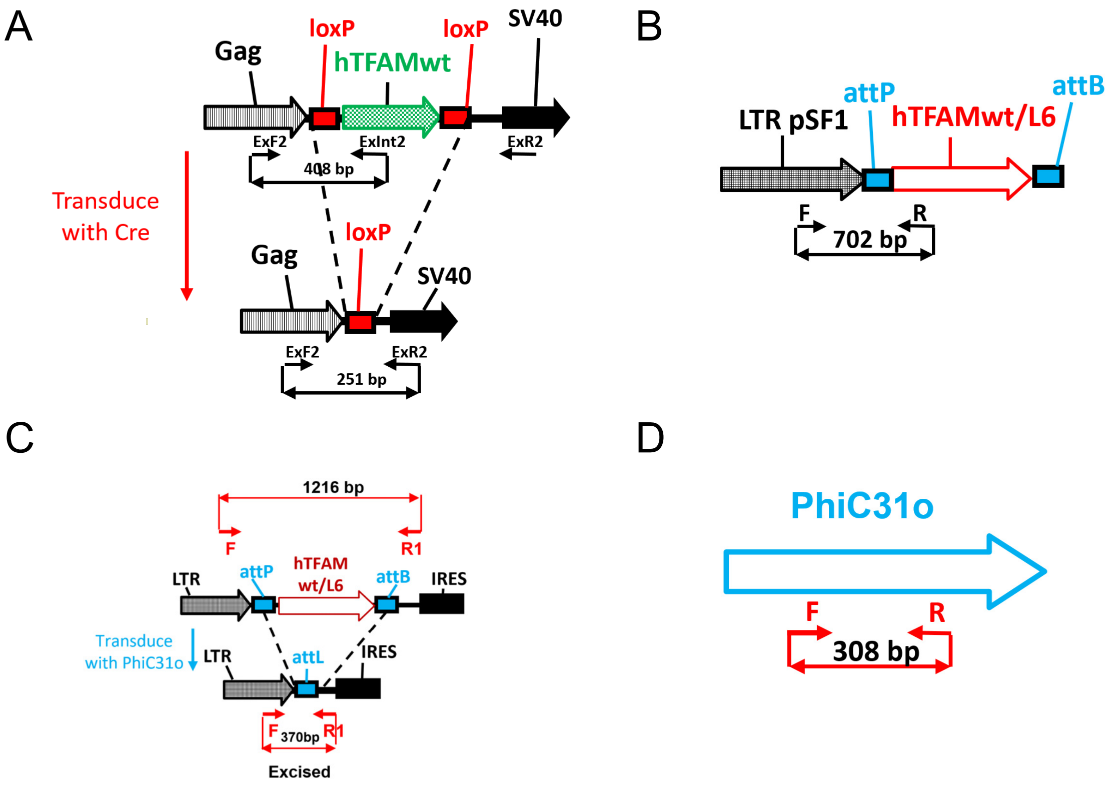

Figure S2. Diagrams for validating substitution of hTFAMwt or hTFAM L6 for hTFAM wt in 143B#6 cells. A, PCR genotyping of hTFAMwt excision in 143B#6 cells. B, PCR genotyping of transduction of 143B cells with either hTFAMwt or hTFAMwt L6. C, PCR genotyping of excision of hTFAMwt and hTFAM L6 by transduction with PhiC31o recombinase. D, PCR genotyping of transduction with PhiC31o recombinase. Designations: G

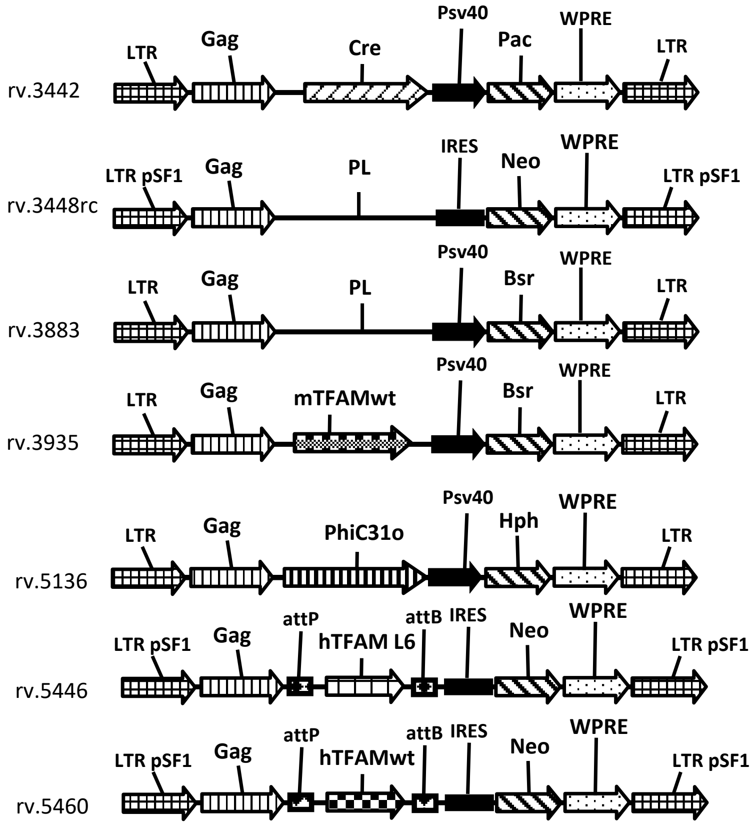

Figure S3. Maps of retroviral constructs used in this study. Designations: attP, attB, recombination sites for PhiC31 recombinase; Cre, gene encoding the bacteriophage P1 Cre recombinase; Gag, truncated retroviral Gag protein; Hph, hygromycin phosphotransferase, hygromycin resistance gene; hTFAMwt, wild-type hTFAM; IRES, internal ribosome entry site; LTR, MoMuLV retroviral long terminal repeat; LTR pSF1, pSF1-derived hybrid retroviral LTR; Neo, G418 and kanamycin resistance gene; Pac, puromycin resistance gene; PhiC31o, optimized PhiC31 recombinase gene; PL, polylinker; SV40, promoter of the SV40 virus; WPRE, woodchuck hepatitis virus posttranscriptional regulatory element.

The utility of the vectors is as follows:

rv.3442 (Addgene#184852). A retroviral vector encoding Cre recombinase and puromycin resistance. Used to deliver Cre recombinase to effect wt hTFAM excision in 143B#6 cells.

rv.3448rc. A retroviral vector for the delivery of wt and truncated hTFAM variants. Encodes G418 resistance.

rv.3883. A retroviral vector for the delivery of mTFAM variants. Encodes blasticidin resistance.

rv.3935. A retroviral vector for the delivery of mTFAMwt. Encodes blasticidin resistance. Vectors for the delivery of truncated mTFAM variants have identical organization.

rv.5136 (Addgene#184853). A retroviral vector encoding PhiC31 recombinase and hygromycin resistance. Used to deliver PhiC31o recombinase to effect excision of the L6 hTFAM bending mutant.

rv.5446. A retroviral vector encoding PhiC31-excisable hTFAM L6 mutant and G418 resistance.

rv.5460 (Addgene#184855). A retroviral vector encoding wt hTFAM flanked by PhiC31 attP and attB sites and G418 resistance.
