# Supplementary material for: The C-Terminal Tail of Mitochondrial Transcription Factor A Is Dispensable for Mitochondrial DNA Replication and Transcription In Situ
Source: Int J Mol Sci. 2023 May 29;24(11):9430. doi: 10.3390/ijms24119430 (PMC10253692; doi:10.3390/ijms24119430)
Supplement: Supplementary file 1 [file ijms-24-09430-s001.zip › Table S1 oligos.pdf]

Table S1. Oligonucleotides used in this study.

Purpose	Name	Sequence	Amplicon, bp
**Genotyping**			
Genotyping of the ρ^0 state in human cells	hMitF	AATGTCTGCACAGCCACTTTCCAC	901
	hMitR	TCGTAGTGTTCTGGCGAGCAGTTT	
	hPOLRMTf	AGAATGTGGCAGTGGAGATG	618
	hPOLRMTr	CCAGTCTAAATGAGGGCAAGT	
Genotyping of hTFAM excision in 143B#6 cells	ExF2	CGCCTCAATCCTCCCTTTATC	WT=408 Excised=251
	ExR2	CACACCTGGTTGCTGACTAA	
	ExInt2	GCATCTGGGTTCTGAGCTTTA	
hTFAMΔ diagnostics	F	TTCGTGTCTGTCTCTGTCTTTG	702
	R	GCATCTGGGTTCTGAGCTTTA	
Genotyping the ρ^0 state in murine cells	mMitF	AAAGCATCTGGCCTACACCCAGAA	1041
	mMitR	ACCCTCGTTTAGCCGTTCATGCTA	
	mNucF	CCACGTGCTCTGTATGAGATT	636
	mNucR	ATGCTGGCTTATCTGTTCCTT	
Genotyping mTFAM exons 6+7 deletion	A	CTGCCTTCCTCTAGCCCGGG	
	B	GTAACAGCAGACAACTTGTG	loxP/loxP=437
	Del	CTCTGAAGCACATGGTCAAT	Del=329
Genotyping PhiC31 recombinase	PhiC31F1	AAGCGCCAACGAGGATAAG	308
	PhiC31R1	TCACGTTGCCCTGTCTAAAG	
Genotyping of PhiC31 excision	F	ACCTACCCGAGTCGGACTTT	WT=1216 Excised=370
	R1	GTTATTGCTTGGGATGTACTTGG	
**RT-qPCR of human transcripts**			
hHPRT	hHPRTf	CGAGATGTGATGAAGGAGATGG	N/A
	hHPRTr	TTGATGTAATCCAGCAGGTCAG	
hMT-ND1	hND1F	GAAGTCACCCTAGCCATCATTC	N/A
	hND1R	GCAGGAGTAATCAGAGGTGTTC	
hMT-ND6	hND6F	CCACACCGCTAACAATCAATAC	N/A
	hND6R	GTTTCTGTTGAGTGTGGGTTTAG	
hMT-RNR2 (16S)	h16Sf	GAAACCAGACGAGCTACCTAAG	N/A
	h16Sr	GGTTTGTCGCCTCTACCTATAAA	
hMT-CO1	hCox1F	CTAGCAGGTGTCTCCTCTATCT	N/A
	hCox1R	GGCGTTTGGTATTGGGTTATG	
hTFAMΔ	F	CCTCTCTCCAAGCTCACTTAC	N/A
	R	GCTCTGCTCCAGACCTTC	

**RT-qPCR of murine transcripts**			
mGAPDH	mGAPDHf	GCACAGTCAAGGCCGAGAAT	N/A
	mGAPDHr	GCCTTCTCCATGGTGGTGAA	
mMT-ND5	mND5F	CGGAGCCCTAACCACATTATT	N/A
	mND5R	GCCTAGTTGGCTTGATGTAGAG	
mMT-ND6	mND6F	CACCCAGCTACTACCATCATTC	N/A
	mND6R	GTTTGGGAGATTGGTTGATGTATG	
mMT-RNR2 (16S)	m16Sf	ACTTCTAACTAAAAGAATTACAGC	N/A
	m16Sr	TAGACGAGTTGATTCATAAAATTG	

mMT-CO1	mCO1f	TGTATGAGCCCACCACATATTC	N/A
	mCO1r	CACCGGTAGGAATTGCGATAA	

mtCN determination by dddPCR

mtDNA copy number determination in human cells by ddPCR	NucF	AACTTGTAAGTGGTAGTGCATAGA	N/A
	NucR	GTAGGAGGACATTTGAGGAGTG	
	NucProbe	FAM-TCAGGCAGACTGACACTAGAGTTCACA-BHQ1	
	MitF	CTGATCAGGGTGAGCATCAAA	
	MitR	GAATGATGGCTAGGGTGACTTC	
	MitProbe	Hex-TGCGAGCAGTAGCCCAAACAATCT-BHQ1	
mtDNA copy number determination in murine cells by ddPCR	NucF	CCTGGGCTTTGAACTTGTCTA	N/A
	NucR	TGAGGGCATTGGAGATTGTG	
	NucProbe	FAM-TGGTCCTGCTATCAGAGATGCAACG-BHQ1	
	MitF	GGCCTATTAATCGCAGCTACAG	
	MitR	GTAGTGCTGAAACTGGTGTAGG	
	MitProbe	FAM-ATTTGGCCTCCACCCATGACTACC-BHQ1	
